# Bis[2-(benzyl­amino)pyridine-κ*N*](2-formyl-6-methoxy­phenolato-κ^2^ *O* ^1^,*O* ^6^)(nitrato-κ^2^ *O*,*O*′)nickel(II)

**DOI:** 10.1107/S1600536809035570

**Published:** 2009-09-09

**Authors:** Ray J. Butcher, Yilma Gultneh, Kouassi Ayikoé

**Affiliations:** a Department of Chemistry, Howard University, 525 College Street NW, Washington, DC 20059, USA

## Abstract

In the title compound, [Ni(C_8_H_7_O_3_)(NO_3_)(C_12_H_12_N_2_)_2_], the asymmetric unit contains a Ni^II^ atom, two mol­ecules of 2-(benzyl­amino)pyridine, a mol­ecule of deprotonated *o*-vanillin (3-methoxy­salicylaldehydate) and a bidentate nitrate anion. The Ni^II^ center is six-coordinated by two pyridine N atoms from 2-(benzyl­amino)pyridine, two O atoms from *o*-vanillin and two O atoms from the nitrate anion. The crystal packing shows two hydrogen bonds from the amine N—H group to the deprotonated phenol O atom of the *o*-vanillin moieties, as well as weak C—H⋯O secondary inter­actions. These inter­actions link the mol­ecules into ribbons in the *c* direction. The steric requirement of the bidentate nitrate and its small bite angle [61.01 (3)°] cause some orientation of the two 2-(benzyl­amino)pyridine groups. As a result, this coordination environment of the Ni^II^ center is distorted octa­hedral, as the *trans* angles range from 158.65 (3) to 175.76 (3)° and the *cis* angles range from 61.01 (3) (for the bidentate nitrate O atoms) to 102.30 (4)°.

## Related literature

For our continuing studies of nickel-containing metalloenzymes, see: Gultneh *et al.* (2008 ▶). For literature related to mixed ligand nitrato complexes of Ni, see: Fernández-Fernández *et al.* (2006 ▶); Tokii *et al.* (1979 ▶). For literature related to the catalytic activity of mixed ligand complexes of nickel, see: Gao *et al.* (2008 ▶). For a description of the Cambridge Structural Database, see: Allen (2002 ▶).
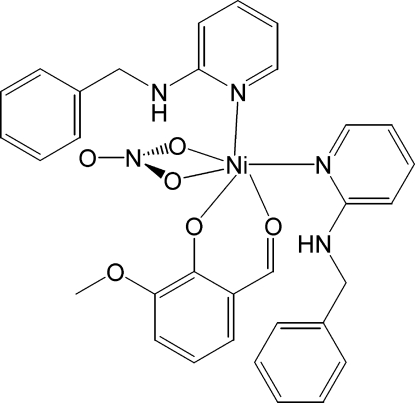


## Experimental

### 

#### Crystal data

[Ni(C_8_H_7_O_3_)(NO_3_)(C_12_H_12_N_2_)_2_]
*M* *_r_* = 640.33
Monoclinic, 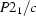

*a* = 10.3522 (2) Å
*b* = 16.7539 (3) Å
*c* = 16.8132 (3) Å
β = 95.5831 (17)°
*V* = 2902.25 (9) Å^3^
*Z* = 4
Mo *K*α radiation
μ = 0.72 mm^−1^
*T* = 110 K
0.48 × 0.41 × 0.22 mm

#### Data collection

Oxford Diffraction Xcalibur diffractometer with a Ruby (Gemini Mo) detector
Absorption correction: multi-scan (*CrysAlisPro*; Oxford Diffraction, 2009 ▶) *T*_min_ = 0.724, *T*_max_ = 0.861
21278 measured reflections
9636 independent reflections
6913 reflections with *I* > 2σ(*I*)
*R*_int_ = 0.024

#### Refinement

*R*[*F* ^2^ > 2σ(*F* ^2^)] = 0.031
*wR*(*F* ^2^) = 0.071
*S* = 0.92
9636 reflections
404 parameters
H atoms treated by a mixture of independent and constrained refinement
Δρ_max_ = 0.37 e Å^−3^
Δρ_min_ = −0.42 e Å^−3^

### 

Data collection: *CrysAlisPro* (Oxford Diffraction, 2009 ▶); cell refinement: *CrysAlisPro*; data reduction: *CrysAlisPro*; program(s) used to solve structure: *SHELXS97* (Sheldrick, 2008 ▶); program(s) used to refine structure: *SHELXL97* (Sheldrick, 2008 ▶); molecular graphics: *SHELXTL* (Sheldrick, 2008 ▶); software used to prepare material for publication: *SHELXTL*.

## Supplementary Material

Crystal structure: contains datablocks I, global. DOI: 10.1107/S1600536809035570/nc2156sup1.cif

Structure factors: contains datablocks I. DOI: 10.1107/S1600536809035570/nc2156Isup2.hkl

Additional supplementary materials:  crystallographic information; 3D view; checkCIF report

## Figures and Tables

**Table 1:** Selected geometric parameters (Å, °)

Ni—O1*A*	1.9690 (8)
Ni—N1*B*	2.0555 (10)
Ni—O2*A*	2.0565 (8)
Ni—O1	2.1148 (8)
Ni—N1*C*	2.1230 (9)
Ni—O2	2.1476 (9)

**Table 2:** Hydrogen-bond geometry (Å, °)

*D*—H⋯*A*	*D*—H	H⋯*A*	*D*⋯*A*	*D*—H⋯*A*
C3*A*—H3*AC*⋯O1^i^	0.98	2.57	3.3537 (15)	137
C4*A*—H4*AA*⋯O1^i^	0.95	2.55	3.4357 (15)	155
C4*B*—H4*BA*⋯O2^ii^	0.95	2.54	3.4308 (15)	157
C6*B*—H6*BB*⋯O2^ii^	0.99	2.57	3.4670 (17)	151
N2*B*—H2*BN*⋯O1*A*	0.893 (15)	2.088 (15)	2.9215 (14)	154.9 (12)
N2*C*—H2*CN*⋯O1*A*	0.754 (14)	2.056 (14)	2.7655 (13)	156.8 (16)
N2*C*—H2*CN*⋯O3*A*	0.754 (14)	2.669 (14)	3.2124 (13)	130.8 (13)

Symmetry codes: (i) 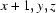; (ii) 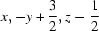.

**Fig. 1.**
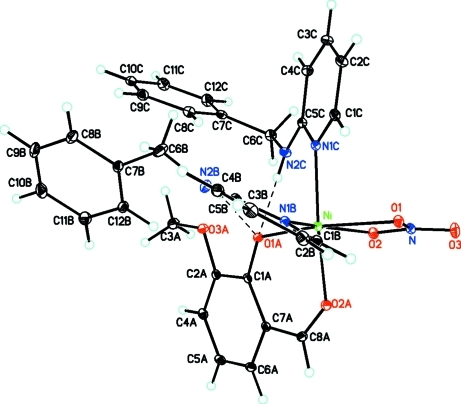
The molecular structure of the dinuclear complex, C32H31N5NiO6 showing the atom numbering scheme and 50% probability displacement ellipsoids.

**Fig. 2.**
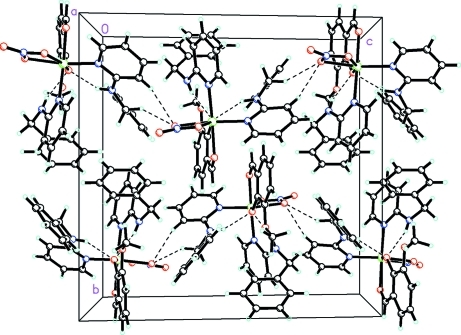
The molecular packing for C32H31N5NiO6, viewed down the a axis showing the intermolecular N—H···O and C—H···O interactions.

## supplementary crystallographic information

### Comment

As part of our continuing studies (Gultneh *et al.* 2008) of nickel(II) complexes with relevance to the nickel containing metalloenzymes we wish to report the structure of the mixed ligand complex, bis(2-(benzylamino)pyridine-κ*N*)(3-methoxysalicylaldehydo- κ^2^*O*,*O*')nitrato-κ^2^*O*,*O*' nickel(II). The title compound, C_32_H_31_N_5_NiO_6_, contains two 2-(benzylamino)pyridine ligands (2-BAP), a bidentate nitrate anion, and a deprotonated *o*-vanillin moiety coordinated to nickel. The nickel atom is six coordinated by two pyridine N atoms from 2-(benzylamino)pyridine, two O from *o*-vanillin and two O atoms from the nitrate anion. Thus in the title complex, the 2-BAP (2 molecules) coordinate to Ni individually forming pendant arms that render the structure flexible. Similar mixed ligand complexes have been synthesized (Fernández-Fernández *et al.* 2006), however, in this case, the nitrate coordinated to the metal through only one O donor atom as a monodentate ligand. 2-Aminopyridine containing *N*-aryl subsituents (a ligand with both an amine donor and a pyridine donor similar to the donors in 2-BAP) has been used (Gao *et al.* 2008) along with halogens such as bromide, to synthesize a series nickel(II) complexes with potential use as precatalysts for ethylene polymerization. A combination of bidentate nitrate ions and tridentate Schiff bases have been used to synthesize dinuclear nickel complex with ligands derived from salicylaldehydes and N-substituted trimethylenediamines (Tokii *et al.*, 1979).

The Ni—O (nitrate) bond distances (see Table 1) [Ni—O(1), 2.1148 (8) Å; Ni—O(2), 2.1476 (9) Å], Ni—O (*o*-vanillin) bond distances [Ni—O(1 A), 1.9690 (8) Å; Ni—O(2 A), 2.0565 (8) Å] and Ni—N (2-BAP) bond distances [Ni—N(1B), 2.0555 (10) Å; Ni—N(1 C), 2.1230 (9) Å] are within the normal ranges observed in other Ni complexes containing similar ligands (Allen, 2002). The geometry about the central Ni is distorted octahedral due to the small bite angle (see Table 1) subtended by the bidentate nitrate anion (O1—Ni—O2, 61.01 (3)°). This causes some re-orientation of the two 2-(benzylamino)pyridine groups. As a result, this coordination environment of the Ni is distorted octahedral as the *trans* angles range from 158.65 (3)° to 175.76 (3)° and the *cis* angles range from 61.01 (3)° (for the bidentate nitrate anion O's) to 102.30 (4)°.

N—H···O hydrogen bonds and weak C—H···O secondary interactions link the molecules into ribbons in the c direction (see Table 2).

### Experimental

The complex was synthesized by reacting 0.73 g (2.0 mmol) of Ni(NO_3_)_2_.6H_2_O in MeOH (20 ml) with a mixture of 0.302 g *o*-vanillin (2 mmol) and 0.370 g of 2-(benzylamino)pyridine (2 mmol). The secondary amine and the aldehyde were mixed in 30 mL of methanol (MeOH) and stirred overnight at 40 C. The solution of the salt and the two ligands was stirred overnight at room temperature. The mixture was evaporated under reduced pressure and dark green semi-solid was obtained. The solid was then dissolved in 50/50 MeOH/DMF. The solution obtained was filtered and layered with diethyl ether. Light yellow greenish X-ray quality crystals were obtained after slow diffusion of the diethyl ether into the MeOH/DMF solution.

### Refinement

H atoms were placed in geometrically idealized positions and constrained to ride on their parent atoms with a C—H distance of 0.95 to 0.99 Å and *U*_iso_(H) = 1.2*U*_eq_(C) [*U*_iso_(H) = 1.5*U*_eq_(C) for the CH_3_]. The positional parameters for the H atoms attached to N were refined with *U*_iso_(H) = 1.2*U*_eq_(N).

### Crystal data

**Table:**

[Ni(C_8_H_7_O_3_)(NO_3_)(C_12_H_12_N_2_)_2_]	*F*(000) = 1336
*M**_r_* = 640.33	*D*_x_ = 1.465 Mg m^−^^3^
Monoclinic, *P*2_1_/*c*	Mo *K*α radiation, λ = 0.71073 Å
*a* = 10.3522 (2) Å	Cell parameters from 10239 reflections
*b* = 16.7539 (3) Å	θ = 4.6–32.7°
*c* = 16.8132 (3) Å	µ = 0.72 mm^−^^1^
β = 95.5831 (17)°	*T* = 110 K
*V* = 2902.25 (9) Å^3^	Plate, deep green
*Z* = 4	0.48 × 0.41 × 0.22 mm

### Data collection

**Table:**

Oxford Diffraction Xcalibur with a Ruby (Gemini Mo) detector diffractometer	9636 independent reflections
Radiation source: Enhance (Mo) X-ray Source	6913 reflections with *I* > 2σ(*I*)
graphite	*R*_int_ = 0.024
Detector resolution: 10.5081 pixels mm^-1^	θ_max_ = 32.7°, θ_min_ = 4.7°
ω scans	*h* = −15→11
Absorption correction: multi-scan (*CrysAlis PRO*; Oxford Diffraction, 2009)	*k* = −25→23
*T*_min_ = 0.724, *T*_max_ = 0.861	*l* = −25→22
21278 measured reflections	

### Refinement

**Table:**

Refinement on *F*^2^	Primary atom site location: structure-invariant direct methods
Least-squares matrix: full	Secondary atom site location: difference Fourier map
*R*[*F*^2^ > 2σ(*F*^2^)] = 0.031	Hydrogen site location: inferred from neighbouring sites
*wR*(*F*^2^) = 0.071	H atoms treated by a mixture of independent and constrained refinement
*S* = 0.92	*w* = 1/[σ^2^(*F*_o_^2^) + (0.0363*P*)^2^] where *P* = (*F*_o_^2^ + 2*F*_c_^2^)/3
9636 reflections	(Δ/σ)_max_ = 0.003
404 parameters	Δρ_max_ = 0.37 e Å^−^^3^
0 restraints	Δρ_min_ = −0.42 e Å^−^^3^

### Special details

**Table:**

Experimental. CrysAlisPro, Oxford Diffraction Ltd., Version 1.171.33.34d (release 27-02-2009 CrysAlis171 .NET) (compiled Feb 27 2009,15:38:38) Empirical absorption correction using spherical harmonics, implemented in SCALE3 ABSPACK scaling algorithm. (Oxford Diffraction, 2008)
Geometry. All e.s.d.'s (except the e.s.d. in the dihedral angle between two l.s. planes) are estimated using the full covariance matrix. The cell e.s.d.'s are taken into account individually in the estimation of e.s.d.'s in distances, angles and torsion angles; correlations between e.s.d.'s in cell parameters are only used when they are defined by crystal symmetry. An approximate (isotropic) treatment of cell e.s.d.'s is used for estimating e.s.d.'s involving l.s. planes.
Refinement. Refinement of *F*^2^ against ALL reflections. The weighted *R*-factor *wR* and goodness of fit *S* are based on *F*^2^, conventional *R*-factors *R* are based on *F*, with *F* set to zero for negative *F*^2^. The threshold expression of *F*^2^ > σ(*F*^2^) is used only for calculating *R*-factors(gt) *etc*. and is not relevant to the choice of reflections for refinement. *R*-factors based on *F*^2^ are statistically about twice as large as those based on *F*, and *R*- factors based on ALL data will be even larger.

### Fractional atomic coordinates and isotropic or equivalent isotropic displacement parameters (Å^2^)

**Table:**

	*x*	*y*	*z*	*U*_iso_*/*U*_eq_	
Ni	0.202156 (14)	0.653615 (8)	0.569477 (9)	0.01381 (4)	
O1	0.02030 (8)	0.63205 (5)	0.61280 (5)	0.01873 (17)	
O2	0.19371 (8)	0.63743 (5)	0.69558 (5)	0.01888 (18)	
O3	0.00620 (9)	0.61203 (6)	0.74021 (5)	0.0285 (2)	
O1A	0.39179 (8)	0.66419 (4)	0.57083 (5)	0.01595 (17)	
O2A	0.21780 (8)	0.53148 (5)	0.56286 (5)	0.01781 (17)	
O3A	0.62621 (8)	0.71334 (4)	0.61510 (5)	0.01908 (18)	
N	0.07114 (10)	0.62649 (6)	0.68502 (6)	0.0184 (2)	
N1B	0.14274 (9)	0.65492 (5)	0.44922 (6)	0.01552 (19)	
N2B	0.30972 (10)	0.73612 (6)	0.41582 (6)	0.0209 (2)	
H2BN	0.3426 (14)	0.7295 (8)	0.4665 (9)	0.025*	
N1C	0.17410 (10)	0.77842 (6)	0.58129 (6)	0.0164 (2)	
N2C	0.36709 (10)	0.80155 (6)	0.65929 (7)	0.0202 (2)	
H2CN	0.3901 (14)	0.7620 (8)	0.6447 (9)	0.024*	
C1A	0.47770 (11)	0.60855 (6)	0.59056 (7)	0.0142 (2)	
C2A	0.60871 (11)	0.63213 (7)	0.61289 (7)	0.0157 (2)	
C3A	0.75547 (12)	0.74125 (7)	0.63705 (9)	0.0252 (3)	
H3AA	0.7832	0.7250	0.6921	0.038*	
H3AB	0.7574	0.7996	0.6332	0.038*	
H3AC	0.8143	0.7183	0.6009	0.038*	
C4A	0.70497 (12)	0.57673 (7)	0.63104 (7)	0.0188 (2)	
H4AA	0.7919	0.5939	0.6442	0.023*	
C5A	0.67562 (12)	0.49474 (7)	0.63028 (7)	0.0202 (3)	
H5AA	0.7424	0.4568	0.6435	0.024*	
C6A	0.55120 (12)	0.46981 (7)	0.61059 (7)	0.0185 (2)	
H6AA	0.5320	0.4143	0.6098	0.022*	
C7A	0.45020 (11)	0.52568 (6)	0.59123 (7)	0.0149 (2)	
C8A	0.32180 (12)	0.49455 (7)	0.57327 (7)	0.0174 (2)	
H8AA	0.3155	0.4381	0.5687	0.021*	
C1B	0.03721 (12)	0.60977 (7)	0.42644 (8)	0.0191 (2)	
H1BA	0.0027	0.5774	0.4657	0.023*	
C2B	−0.02289 (12)	0.60799 (8)	0.35011 (8)	0.0231 (3)	
H2BA	−0.0960	0.5748	0.3364	0.028*	
C3B	0.02721 (13)	0.65673 (7)	0.29319 (8)	0.0246 (3)	
H3BA	−0.0136	0.6583	0.2401	0.030*	
C4B	0.13525 (13)	0.70228 (7)	0.31391 (7)	0.0220 (3)	
H4BA	0.1692	0.7358	0.2754	0.026*	
C5B	0.19575 (11)	0.69900 (7)	0.39280 (7)	0.0167 (2)	
C6B	0.36416 (13)	0.80128 (8)	0.37277 (10)	0.0317 (3)	
H6BA	0.3370	0.8524	0.3956	0.038*	
H6BB	0.3268	0.7993	0.3163	0.038*	
C7B	0.50969 (12)	0.80081 (7)	0.37477 (7)	0.0185 (2)	
C8B	0.56829 (14)	0.85753 (7)	0.32841 (8)	0.0255 (3)	
H8BA	0.5159	0.8945	0.2969	0.031*	
C9B	0.70163 (14)	0.86001 (8)	0.32834 (8)	0.0302 (3)	
H9BA	0.7401	0.8991	0.2972	0.036*	
C10B	0.77964 (14)	0.80634 (8)	0.37307 (9)	0.0314 (3)	
H10A	0.8712	0.8077	0.3719	0.038*	
C11B	0.72309 (13)	0.75061 (8)	0.41959 (8)	0.0274 (3)	
H11A	0.7760	0.7138	0.4510	0.033*	
C12B	0.58900 (12)	0.74829 (7)	0.42047 (7)	0.0207 (3)	
H12A	0.5512	0.7101	0.4529	0.025*	
C1C	0.05938 (12)	0.80597 (7)	0.54716 (7)	0.0200 (2)	
H1CA	0.0048	0.7697	0.5162	0.024*	
C2C	0.01632 (13)	0.88294 (7)	0.55423 (8)	0.0256 (3)	
H2CA	−0.0657	0.8994	0.5293	0.031*	
C3C	0.09726 (14)	0.93606 (7)	0.59929 (8)	0.0271 (3)	
H3CA	0.0703	0.9896	0.6060	0.033*	
C4C	0.21554 (13)	0.91095 (7)	0.63380 (8)	0.0234 (3)	
H4CA	0.2718	0.9472	0.6637	0.028*	
C5C	0.25326 (12)	0.83049 (7)	0.62454 (7)	0.0175 (2)	
C6C	0.46296 (12)	0.84853 (7)	0.70677 (7)	0.0215 (2)	
H6CA	0.5180	0.8119	0.7416	0.026*	
H6CB	0.4172	0.8839	0.7420	0.026*	
C7C	0.55110 (12)	0.89975 (7)	0.66056 (7)	0.0182 (2)	
C8C	0.54335 (13)	0.90071 (7)	0.57792 (8)	0.0216 (3)	
H8CA	0.4776	0.8707	0.5480	0.026*	
C9C	0.63066 (13)	0.94512 (7)	0.53805 (8)	0.0251 (3)	
H9CA	0.6248	0.9450	0.4813	0.030*	
C10C	0.72632 (13)	0.98958 (7)	0.58141 (9)	0.0262 (3)	
H10B	0.7858	1.0202	0.5544	0.031*	
C11C	0.73488 (14)	0.98918 (8)	0.66394 (9)	0.0289 (3)	
H11B	0.8006	1.0193	0.6938	0.035*	
C12C	0.64761 (13)	0.94482 (8)	0.70305 (8)	0.0264 (3)	
H12B	0.6536	0.9451	0.7598	0.032*	

### Atomic displacement parameters (Å^2^)

**Table:**

	*U*^11^	*U*^22^	*U*^33^	*U*^12^	*U*^13^	*U*^23^
Ni	0.01023 (7)	0.01481 (7)	0.01645 (7)	0.00088 (6)	0.00155 (5)	0.00061 (6)
O1	0.0134 (4)	0.0241 (4)	0.0185 (4)	0.0016 (3)	0.0008 (3)	0.0026 (3)
O2	0.0127 (4)	0.0226 (4)	0.0211 (4)	0.0008 (3)	0.0010 (3)	0.0001 (3)
O3	0.0223 (5)	0.0413 (5)	0.0234 (5)	0.0010 (4)	0.0104 (4)	0.0052 (4)
O1A	0.0111 (4)	0.0145 (4)	0.0221 (4)	0.0022 (3)	0.0011 (3)	0.0023 (3)
O2A	0.0153 (4)	0.0168 (4)	0.0215 (4)	−0.0003 (3)	0.0029 (3)	0.0001 (3)
O3A	0.0108 (4)	0.0159 (4)	0.0300 (5)	−0.0005 (3)	−0.0009 (3)	0.0018 (3)
N	0.0152 (5)	0.0192 (5)	0.0212 (5)	0.0023 (4)	0.0036 (4)	0.0009 (4)
N1B	0.0122 (5)	0.0159 (4)	0.0186 (5)	0.0014 (4)	0.0025 (4)	−0.0003 (4)
N2B	0.0193 (6)	0.0219 (5)	0.0215 (5)	−0.0045 (4)	0.0014 (4)	0.0043 (4)
N1C	0.0143 (5)	0.0167 (5)	0.0185 (5)	0.0012 (4)	0.0029 (4)	0.0009 (4)
N2C	0.0180 (5)	0.0170 (5)	0.0253 (6)	0.0014 (4)	0.0003 (4)	−0.0052 (4)
C1A	0.0130 (5)	0.0173 (5)	0.0126 (5)	0.0034 (5)	0.0034 (4)	0.0007 (4)
C2A	0.0136 (6)	0.0181 (5)	0.0157 (5)	0.0013 (5)	0.0028 (4)	0.0016 (4)
C3A	0.0131 (6)	0.0229 (6)	0.0389 (8)	−0.0017 (5)	−0.0007 (5)	0.0029 (6)
C4A	0.0130 (6)	0.0234 (6)	0.0199 (6)	0.0023 (5)	0.0011 (5)	0.0003 (5)
C5A	0.0187 (6)	0.0204 (6)	0.0213 (6)	0.0087 (5)	0.0013 (5)	0.0007 (5)
C6A	0.0225 (6)	0.0157 (5)	0.0176 (6)	0.0041 (5)	0.0030 (5)	0.0003 (4)
C7A	0.0161 (6)	0.0159 (5)	0.0132 (5)	0.0027 (5)	0.0035 (4)	0.0003 (4)
C8A	0.0215 (6)	0.0144 (5)	0.0167 (6)	−0.0004 (5)	0.0042 (5)	0.0004 (4)
C1B	0.0149 (6)	0.0204 (6)	0.0221 (6)	−0.0002 (5)	0.0026 (5)	−0.0010 (5)
C2B	0.0171 (6)	0.0275 (6)	0.0245 (7)	−0.0018 (5)	0.0004 (5)	−0.0047 (5)
C3B	0.0231 (7)	0.0307 (7)	0.0191 (6)	0.0052 (6)	−0.0025 (5)	−0.0018 (5)
C4B	0.0258 (7)	0.0218 (6)	0.0187 (6)	0.0023 (5)	0.0042 (5)	0.0009 (5)
C5B	0.0146 (6)	0.0157 (5)	0.0202 (6)	0.0032 (5)	0.0036 (5)	−0.0010 (5)
C6B	0.0232 (7)	0.0243 (6)	0.0471 (9)	−0.0016 (6)	0.0009 (6)	0.0174 (6)
C7B	0.0218 (6)	0.0172 (5)	0.0167 (6)	−0.0049 (5)	0.0037 (5)	−0.0026 (5)
C8B	0.0331 (8)	0.0228 (6)	0.0209 (6)	−0.0066 (6)	0.0049 (5)	0.0023 (5)
C9B	0.0355 (8)	0.0312 (7)	0.0260 (7)	−0.0129 (6)	0.0139 (6)	−0.0010 (6)
C10B	0.0227 (7)	0.0376 (8)	0.0355 (8)	−0.0064 (6)	0.0108 (6)	−0.0058 (6)
C11B	0.0222 (7)	0.0289 (7)	0.0311 (7)	−0.0008 (6)	0.0022 (6)	−0.0003 (6)
C12B	0.0234 (7)	0.0193 (6)	0.0200 (6)	−0.0037 (5)	0.0047 (5)	−0.0004 (5)
C1C	0.0149 (6)	0.0224 (6)	0.0230 (6)	0.0020 (5)	0.0031 (5)	0.0019 (5)
C2C	0.0214 (7)	0.0245 (6)	0.0315 (7)	0.0079 (5)	0.0054 (6)	0.0048 (6)
C3C	0.0311 (8)	0.0180 (6)	0.0339 (8)	0.0083 (6)	0.0116 (6)	0.0036 (5)
C4C	0.0257 (7)	0.0174 (6)	0.0280 (7)	−0.0002 (5)	0.0070 (5)	−0.0018 (5)
C5C	0.0176 (6)	0.0171 (5)	0.0185 (6)	0.0004 (5)	0.0054 (5)	0.0012 (4)
C6C	0.0223 (6)	0.0233 (6)	0.0184 (6)	−0.0018 (5)	−0.0002 (5)	−0.0033 (5)
C7C	0.0182 (6)	0.0148 (5)	0.0215 (6)	0.0021 (5)	0.0012 (5)	−0.0030 (5)
C8C	0.0209 (6)	0.0226 (6)	0.0206 (6)	0.0016 (5)	−0.0018 (5)	−0.0025 (5)
C9C	0.0258 (7)	0.0263 (6)	0.0235 (6)	0.0066 (6)	0.0039 (5)	0.0032 (5)
C10C	0.0247 (7)	0.0180 (6)	0.0376 (8)	0.0026 (5)	0.0110 (6)	0.0010 (5)
C11C	0.0257 (7)	0.0261 (6)	0.0353 (8)	−0.0074 (6)	0.0048 (6)	−0.0118 (6)
C12C	0.0281 (7)	0.0283 (7)	0.0228 (7)	−0.0041 (6)	0.0025 (5)	−0.0085 (5)

### Geometric parameters (Å, °)

**Table:**

Ni—O1A	1.9690 (8)	C3B—C4B	1.3708 (18)
Ni—N1B	2.0555 (10)	C3B—H3BA	0.9500
Ni—O2A	2.0565 (8)	C4B—C5B	1.4117 (17)
Ni—O1	2.1148 (8)	C4B—H4BA	0.9500
Ni—N1C	2.1230 (9)	C6B—C7B	1.5037 (18)
Ni—O2	2.1476 (9)	C6B—H6BA	0.9900
O1—N	1.2790 (13)	C6B—H6BB	0.9900
O2—N	1.2772 (13)	C7B—C12B	1.3842 (17)
O3—N	1.2218 (13)	C7B—C8B	1.4038 (16)
O1A—C1A	1.3086 (13)	C8B—C9B	1.381 (2)
O2A—C8A	1.2393 (14)	C8B—H8BA	0.9500
O3A—C2A	1.3727 (13)	C9B—C10B	1.381 (2)
O3A—C3A	1.4315 (15)	C9B—H9BA	0.9500
N1B—C1B	1.3532 (15)	C10B—C11B	1.3839 (19)
N1B—C5B	1.3594 (15)	C10B—H10A	0.9500
N2B—C5B	1.3563 (15)	C11B—C12B	1.3901 (18)
N2B—C6B	1.4536 (16)	C11B—H11A	0.9500
N2B—H2BN	0.893 (15)	C12B—H12A	0.9500
N1C—C1C	1.3491 (15)	C1C—C2C	1.3735 (17)
N1C—C5C	1.3585 (15)	C1C—H1CA	0.9500
N2C—C5C	1.3532 (16)	C2C—C3C	1.394 (2)
N2C—C6C	1.4448 (16)	C2C—H2CA	0.9500
N2C—H2CN	0.754 (14)	C3C—C4C	1.3692 (19)
C1A—C7A	1.4176 (15)	C3C—H3CA	0.9500
C1A—C2A	1.4273 (16)	C4C—C5C	1.4163 (16)
C2A—C4A	1.3741 (17)	C4C—H4CA	0.9500
C3A—H3AA	0.9800	C6C—C7C	1.5200 (17)
C3A—H3AB	0.9800	C6C—H6CA	0.9900
C3A—H3AC	0.9800	C6C—H6CB	0.9900
C4A—C5A	1.4066 (17)	C7C—C8C	1.3840 (17)
C4A—H4AA	0.9500	C7C—C12C	1.3928 (18)
C5A—C6A	1.3639 (17)	C8C—C9C	1.3920 (18)
C5A—H5AA	0.9500	C8C—H8CA	0.9500
C6A—C7A	1.4174 (16)	C9C—C10C	1.3880 (19)
C6A—H6AA	0.9500	C9C—H9CA	0.9500
C7A—C8A	1.4325 (17)	C10C—C11C	1.3818 (19)
C8A—H8AA	0.9500	C10C—H10B	0.9500
C1B—C2B	1.3709 (17)	C11C—C12C	1.3845 (19)
C1B—H1BA	0.9500	C11C—H11B	0.9500
C2B—C3B	1.3958 (18)	C12C—H12B	0.9500
C2B—H2BA	0.9500		
			
O1A—Ni—N1B	102.30 (4)	C2B—C3B—H3BA	120.0
O1A—Ni—O2A	90.37 (3)	C3B—C4B—C5B	119.57 (12)
N1B—Ni—O2A	88.57 (3)	C3B—C4B—H4BA	120.2
O1A—Ni—O1	158.65 (3)	C5B—C4B—H4BA	120.2
N1B—Ni—O1	98.59 (4)	N2B—C5B—N1B	116.67 (11)
O2A—Ni—O1	85.76 (3)	N2B—C5B—C4B	122.99 (11)
O1A—Ni—N1C	93.19 (3)	N1B—C5B—C4B	120.30 (11)
N1B—Ni—N1C	92.92 (4)	N2B—C6B—C7B	114.90 (11)
O2A—Ni—N1C	175.76 (3)	N2B—C6B—H6BA	108.5
O1—Ni—N1C	90.09 (3)	C7B—C6B—H6BA	108.5
O1A—Ni—O2	97.83 (3)	N2B—C6B—H6BB	108.5
N1B—Ni—O2	159.29 (4)	C7B—C6B—H6BB	108.5
O2A—Ni—O2	86.49 (3)	H6BA—C6B—H6BB	107.5
O1—Ni—O2	61.01 (3)	C12B—C7B—C8B	118.26 (12)
N1C—Ni—O2	90.74 (3)	C12B—C7B—C6B	123.69 (11)
N—O1—Ni	92.38 (6)	C8B—C7B—C6B	118.05 (11)
N—O2—Ni	90.93 (6)	C9B—C8B—C7B	120.45 (13)
C1A—O1A—Ni	126.23 (7)	C9B—C8B—H8BA	119.8
C8A—O2A—Ni	124.12 (8)	C7B—C8B—H8BA	119.8
C2A—O3A—C3A	116.64 (9)	C8B—C9B—C10B	120.75 (12)
O3—N—O2	122.38 (10)	C8B—C9B—H9BA	119.6
O3—N—O1	121.97 (10)	C10B—C9B—H9BA	119.6
O2—N—O1	115.65 (10)	C9B—C10B—C11B	119.33 (13)
C1B—N1B—C5B	118.41 (10)	C9B—C10B—H10A	120.3
C1B—N1B—Ni	115.38 (8)	C11B—C10B—H10A	120.3
C5B—N1B—Ni	126.14 (8)	C10B—C11B—C12B	120.17 (13)
C5B—N2B—C6B	124.80 (11)	C10B—C11B—H11A	119.9
C5B—N2B—H2BN	117.1 (9)	C12B—C11B—H11A	119.9
C6B—N2B—H2BN	116.0 (9)	C7B—C12B—C11B	121.02 (11)
C1C—N1C—C5C	117.93 (10)	C7B—C12B—H12A	119.5
C1C—N1C—Ni	114.83 (8)	C11B—C12B—H12A	119.5
C5C—N1C—Ni	126.99 (8)	N1C—C1C—C2C	124.33 (12)
C5C—N2C—C6C	124.47 (10)	N1C—C1C—H1CA	117.8
C5C—N2C—H2CN	117.6 (12)	C2C—C1C—H1CA	117.8
C6C—N2C—H2CN	116.0 (12)	C1C—C2C—C3C	117.60 (12)
O1A—C1A—C7A	124.58 (10)	C1C—C2C—H2CA	121.2
O1A—C1A—C2A	118.33 (10)	C3C—C2C—H2CA	121.2
C7A—C1A—C2A	117.09 (10)	C4C—C3C—C2C	119.97 (12)
O3A—C2A—C4A	124.92 (11)	C4C—C3C—H3CA	120.0
O3A—C2A—C1A	113.64 (9)	C2C—C3C—H3CA	120.0
C4A—C2A—C1A	121.43 (10)	C3C—C4C—C5C	119.38 (12)
O3A—C3A—H3AA	109.5	C3C—C4C—H4CA	120.3
O3A—C3A—H3AB	109.5	C5C—C4C—H4CA	120.3
H3AA—C3A—H3AB	109.5	N2C—C5C—N1C	117.11 (10)
O3A—C3A—H3AC	109.5	N2C—C5C—C4C	122.10 (11)
H3AA—C3A—H3AC	109.5	N1C—C5C—C4C	120.77 (11)
H3AB—C3A—H3AC	109.5	N2C—C6C—C7C	116.06 (10)
C2A—C4A—C5A	120.41 (11)	N2C—C6C—H6CA	108.3
C2A—C4A—H4AA	119.8	C7C—C6C—H6CA	108.3
C5A—C4A—H4AA	119.8	N2C—C6C—H6CB	108.3
C6A—C5A—C4A	119.96 (11)	C7C—C6C—H6CB	108.3
C6A—C5A—H5AA	120.0	H6CA—C6C—H6CB	107.4
C4A—C5A—H5AA	120.0	C8C—C7C—C12C	118.51 (12)
C5A—C6A—C7A	120.77 (11)	C8C—C7C—C6C	122.73 (11)
C5A—C6A—H6AA	119.6	C12C—C7C—C6C	118.70 (11)
C7A—C6A—H6AA	119.6	C7C—C8C—C9C	120.85 (12)
C6A—C7A—C1A	120.29 (11)	C7C—C8C—H8CA	119.6
C6A—C7A—C8A	117.17 (10)	C9C—C8C—H8CA	119.6
C1A—C7A—C8A	122.53 (10)	C10C—C9C—C8C	119.83 (12)
O2A—C8A—C7A	128.59 (10)	C10C—C9C—H9CA	120.1
O2A—C8A—H8AA	115.7	C8C—C9C—H9CA	120.1
C7A—C8A—H8AA	115.7	C11C—C10C—C9C	119.83 (12)
N1B—C1B—C2B	123.88 (12)	C11C—C10C—H10B	120.1
N1B—C1B—H1BA	118.1	C9C—C10C—H10B	120.1
C2B—C1B—H1BA	118.1	C10C—C11C—C12C	119.91 (12)
C1B—C2B—C3B	117.65 (12)	C10C—C11C—H11B	120.0
C1B—C2B—H2BA	121.2	C12C—C11C—H11B	120.0
C3B—C2B—H2BA	121.2	C11C—C12C—C7C	121.07 (12)
C4B—C3B—C2B	119.99 (12)	C11C—C12C—H12B	119.5
C4B—C3B—H3BA	120.0	C7C—C12C—H12B	119.5
			
O1A—Ni—O1—N	7.28 (12)	C5A—C6A—C7A—C8A	−178.42 (11)
N1B—Ni—O1—N	175.29 (6)	O1A—C1A—C7A—C6A	177.56 (10)
O2A—Ni—O1—N	87.39 (6)	C2A—C1A—C7A—C6A	−2.26 (16)
N1C—Ni—O1—N	−91.74 (6)	O1A—C1A—C7A—C8A	−2.82 (17)
O2—Ni—O1—N	−0.94 (6)	C2A—C1A—C7A—C8A	177.37 (10)
O1A—Ni—O2—N	−176.04 (6)	Ni—O2A—C8A—C7A	−0.24 (17)
N1B—Ni—O2—N	−9.64 (12)	C6A—C7A—C8A—O2A	171.95 (11)
O2A—Ni—O2—N	−86.15 (6)	C1A—C7A—C8A—O2A	−7.68 (19)
O1—Ni—O2—N	0.94 (6)	C5B—N1B—C1B—C2B	−2.57 (17)
N1C—Ni—O2—N	90.63 (6)	Ni—N1B—C1B—C2B	174.60 (10)
N1B—Ni—O1A—C1A	−109.42 (9)	N1B—C1B—C2B—C3B	−1.00 (18)
O2A—Ni—O1A—C1A	−20.80 (9)	C1B—C2B—C3B—C4B	1.98 (18)
O1—Ni—O1A—C1A	58.45 (13)	C2B—C3B—C4B—C5B	0.50 (18)
N1C—Ni—O1A—C1A	156.89 (9)	C6B—N2B—C5B—N1B	−163.03 (12)
O2—Ni—O1A—C1A	65.70 (9)	C6B—N2B—C5B—C4B	19.34 (19)
O1A—Ni—O2A—C8A	11.28 (9)	C1B—N1B—C5B—N2B	−172.60 (10)
N1B—Ni—O2A—C8A	113.58 (9)	Ni—N1B—C5B—N2B	10.56 (14)
O1—Ni—O2A—C8A	−147.70 (9)	C1B—N1B—C5B—C4B	5.09 (16)
N1C—Ni—O2A—C8A	−135.8 (5)	Ni—N1B—C5B—C4B	−171.74 (8)
O2—Ni—O2A—C8A	−86.54 (9)	C3B—C4B—C5B—N2B	173.40 (11)
Ni—O2—N—O3	178.85 (10)	C3B—C4B—C5B—N1B	−4.15 (17)
Ni—O2—N—O1	−1.51 (9)	C5B—N2B—C6B—C7B	−144.38 (12)
Ni—O1—N—O3	−178.82 (10)	N2B—C6B—C7B—C12B	−6.64 (19)
Ni—O1—N—O2	1.54 (9)	N2B—C6B—C7B—C8B	174.19 (12)
O1A—Ni—N1B—C1B	146.82 (8)	C12B—C7B—C8B—C9B	0.47 (18)
O2A—Ni—N1B—C1B	56.75 (8)	C6B—C7B—C8B—C9B	179.68 (13)
O1—Ni—N1B—C1B	−28.74 (8)	C7B—C8B—C9B—C10B	0.7 (2)
N1C—Ni—N1B—C1B	−119.28 (8)	C8B—C9B—C10B—C11B	−1.3 (2)
O2—Ni—N1B—C1B	−19.40 (14)	C9B—C10B—C11B—C12B	0.7 (2)
O1A—Ni—N1B—C5B	−36.26 (9)	C8B—C7B—C12B—C11B	−1.06 (18)
O2A—Ni—N1B—C5B	−126.33 (9)	C6B—C7B—C12B—C11B	179.78 (13)
O1—Ni—N1B—C5B	148.18 (9)	C10B—C11B—C12B—C7B	0.49 (19)
N1C—Ni—N1B—C5B	57.64 (9)	C5C—N1C—C1C—C2C	−1.05 (18)
O2—Ni—N1B—C5B	157.52 (9)	Ni—N1C—C1C—C2C	173.49 (10)
O1A—Ni—N1C—C1C	150.18 (8)	N1C—C1C—C2C—C3C	0.46 (19)
N1B—Ni—N1C—C1C	47.67 (8)	C1C—C2C—C3C—C4C	0.72 (19)
O2A—Ni—N1C—C1C	−62.8 (5)	C2C—C3C—C4C—C5C	−1.24 (19)
O1—Ni—N1C—C1C	−50.93 (8)	C6C—N2C—C5C—N1C	179.54 (10)
O2—Ni—N1C—C1C	−111.94 (8)	C6C—N2C—C5C—C4C	−1.87 (19)
O1A—Ni—N1C—C5C	−35.87 (10)	C1C—N1C—C5C—N2C	179.08 (10)
N1B—Ni—N1C—C5C	−138.37 (10)	Ni—N1C—C5C—N2C	5.29 (15)
O2A—Ni—N1C—C5C	111.2 (5)	C1C—N1C—C5C—C4C	0.48 (16)
O1—Ni—N1C—C5C	123.02 (10)	Ni—N1C—C5C—C4C	−173.31 (9)
O2—Ni—N1C—C5C	62.02 (10)	C3C—C4C—C5C—N2C	−177.89 (12)
Ni—O1A—C1A—C7A	19.94 (15)	C3C—C4C—C5C—N1C	0.64 (18)
Ni—O1A—C1A—C2A	−160.25 (8)	C5C—N2C—C6C—C7C	−79.83 (15)
C3A—O3A—C2A—C4A	0.89 (17)	N2C—C6C—C7C—C8C	−1.43 (17)
C3A—O3A—C2A—C1A	−179.98 (10)	N2C—C6C—C7C—C12C	−178.40 (11)
O1A—C1A—C2A—O3A	3.61 (15)	C12C—C7C—C8C—C9C	0.57 (18)
C7A—C1A—C2A—O3A	−176.56 (10)	C6C—C7C—C8C—C9C	−176.41 (11)
O1A—C1A—C2A—C4A	−177.22 (11)	C7C—C8C—C9C—C10C	−0.49 (18)
C7A—C1A—C2A—C4A	2.60 (16)	C8C—C9C—C10C—C11C	0.38 (19)
O3A—C2A—C4A—C5A	177.20 (11)	C9C—C10C—C11C—C12C	−0.37 (19)
C1A—C2A—C4A—C5A	−1.86 (18)	C10C—C11C—C12C—C7C	0.5 (2)
C2A—C4A—C5A—C6A	0.70 (18)	C8C—C7C—C12C—C11C	−0.56 (19)
C4A—C5A—C6A—C7A	−0.39 (18)	C6C—C7C—C12C—C11C	176.54 (12)
C5A—C6A—C7A—C1A	1.23 (17)		

### Hydrogen-bond geometry (Å, °)

**Table:**

*D*—H···*A*	*D*—H	H···*A*	*D*···*A*	*D*—H···*A*
C3A—H3AC···O1^i^	0.98	2.57	3.3537 (15)	137
C4A—H4AA···O1^i^	0.95	2.55	3.4357 (15)	155
C4B—H4BA···O2^ii^	0.95	2.54	3.4308 (15)	157
C6B—H6BB···O2^ii^	0.99	2.57	3.4670 (17)	151
N2B—H2BN···O1A	0.893 (15)	2.088 (15)	2.9215 (14)	154.9 (12)
N2C—H2CN···O1A	0.754 (14)	2.056 (14)	2.7655 (13)	156.8 (16)
N2C—H2CN···O3A	0.754 (14)	2.669 (14)	3.2124 (13)	130.8 (13)

Symmetry codes: (i) *x*+1, *y*, *z*; (ii) *x*, −*y*+3/2, *z*−1/2.

## References

[bb1] Allen, F. H. (2002). *Acta Cryst.* B**58**, 380–388. 10.1107/s0108768102003890 12037359

[bb2] Fernández-Fernández, M., Bastida, R., Maćias, A., Valencia, L. & Pérez-Lourido, P. (2006). *Polyhedron*, **25**, 783–792.

[bb3] Gao, H., Huang, Z., Song, K., Liu, F., Long, J., Hu, H. & Wu, Q. (2008). *J. Polym. Sci. Part A Polym. Chem.* **46**, 1618–1628.

[bb4] Gultneh, Y., Khan, A. R., Ahvazi, B. & Butcher, R. J. (2008). *Polyhedron*, **17**, 3351–3360.

[bb5] Oxford Diffraction (2009). *CrysAlisPro* Oxford Diffraction Ltd, Yarnton, Oxfordshire, England.

[bb6] Sheldrick, G. M. (2008). *Acta Cryst.* A**64**, 112–122. 10.1107/S0108767307043930 18156677

[bb7] Tokii, T., Emori, S. & Muto, Y. (1979). *Bull. Chem. Soc. Jpn*, **52**, 2114–2119.

